# Multiple Indicator Cluster Survey 2003 in Afghanistan: Outdated Sampling Frame and the Effect of Sampling Weights on Estimates of Maternal and Child Health Coverage

**DOI:** 10.3329/jhpn.v29i4.8456

**Published:** 2011-08

**Authors:** Shivam Gupta, Muhammad Shuaib, Stan Becker, Md. Mokhlesur Rahman, David H. Peters

**Affiliations:** ^1^Department of International Health, Johns Hopkins Bloomberg School of Public Health, 615 N Wolfe Street, Baltimore, MD 21205, USA; ^2^Institute of Statistical Research and Training, University of Dhaka, Dhaka, Bangladesh,; ^3^Survey Research, Dhaka, Bangladesh

**Keywords:** Maternal health, Child health, Cluster survey, Sampling frame, Sampling weight, Afghanistan

## Abstract

Due to an urgent need for information on the coverage of health service for women and children after the fall of Taliban regime in Afghanistan, a multiple indicator cluster survey (MICS) was conducted in 2003 using the outdated 1979 census as the sampling frame. When 2004 pre-census data became available, population-sampling weights were generated based on the survey-sampling scheme. Using these weights, the population estimates for seven maternal and child healthcare-coverage indicators were generated and compared with the unweighted MICS 2003 estimates. The use of sample weights provided unbiased estimates of population parameters. Results of the comparison of weighted and unweighted estimates showed some wide differences for individual provincial estimates and confidence intervals. However, the mean, median and absolute mean of the differences between weighted and unweighted estimates and their confidence intervals were close to zero for all indicators at the national level. Ranking of the five highest and the five lowest provinces on weighted and unweighted estimates also yielded similar results. The general consistency of results suggests that outdated sampling frames can be appropriate for use in similar situations to obtain initial estimates from household surveys to guide policy and programming directions. However, the power to detect change from these estimates is lower than originally planned, requiring a greater tolerance for error when the data are used as a baseline for evaluation. The generalizability of using outdated sampling frames in similar settings is qualified by the specific characteristics of the MICS 2003—low replacement rate of clusters and zero probability of inclusion of clusters created after the 1979 census.

## INTRODUCTION

The Afghanistan Ministry of Public Health (MoPH) initiated a strategy to reconstruct the health system in 2002 with a focus on laying “the foundations for equitable, quality health care for the people of Afghanistan” (1). The MoPH and other stakeholders required baseline population-level health data for planning and evaluation of this health strategy. Information was particularly needed on the coverage of health services to identify provinces with the greatest problems and to provide a reasonable starting point to gauge future change in the health sector. In the post-Taliban period, the first population-based health survey of national scope was conducted by the United Nations Children's Fund (UNICEF) and the Central Statistics Office (CSO) for the MoPH in 2003. This Multiple Indicator Cluster Survey (MICS) used data of the outdated population census from 1979 for sampling of households. This pragmatic decision was guided by the lack of a national census since 1979 and the urgent need to collect information on the coverage of health services across the country (2). However, questions persisted about the accuracy of the 2003 MICS estimates, given the substantial changes that occurred in the population since the sampling frame was constructed in 1979. An opportunity presented itself to re-assess the 2003 estimates when the CSO conducted a pre-census enumeration in 2004 and, in 2006, published the national and provincial census figures (3).

Population surveys, such as MICS, are important tools for planning, monitoring, and evaluation of health programmes in developing countries. The results of these surveys are used for summative evaluations and for influencing significant policy decisions on allocation of resources, continuation, and restructuring of programmes (4). In recent times, the ‘instrumental’ use of such results has increased as a greater proportion of decisions on programme oversight is directly based on these results (5). The estimates from the MICS 2003 have been put to ‘instrumental’ use as official health indicators for Afghanistan and have been used as benchmarks for health policy (6). Although the MICS 2003 was the first quantitative assessment of coverage of services targeted to women and children in the post-Tali-ban period, a further study was needed to assess whether these estimates would be adequate for providing baseline estimates for future evaluation of healthcare coverage in Afghanistan (7).

The basic approach in population-based surveys is to collect information from a random sample of people that is representative of the population (8). The sampling and data-collection are usually conducted in multiple stages to overcome the constraints of time, money, and logistics. In order for the results to reflect the situation in the population from which the data are collected, the sampling scheme must be incorporated in the analysis. This usually requires the use of sampling weights and statistical techniques to accommodate for the multi-stage sampling design. The purpose of weighting sample data is to assure the representativeness of the sample *vis-a-vis* the study population. The inverse of the selection probability of a sampled unit is used as the sampling weight for that unit. The population estimates generated without sampling weights could be biased (8,9). Evaluations of programmes based on the ‘instrumental’ use of these survey results can be adversely affected by this potential bias and lead to incorrect conclusions. The field of summative evaluation of health programmes can benefit from applied research on this aspect of survey methods. This is especially true in post-conflict settings where the lack of good, routine health information systems, vital registration systems, and census data make household surveys indispensable for information on the health of the population (10). The scarcity of reliable, comprehensive data is considered one of the greatest challenges in planning and evaluating post-conflict reconstruction of the health systems (11).

The clusters for the MICS 2003 were systematically sampled according to the 1979 census using the probability proportional to size (PPS) technique. Therefore, the sample was assumed to be self-weighted, and hence, unweighted estimates for coverage of health services were generated. The present study used the 2004 Afghanistan pre-census figures to generate a set of sampling weights and calculate provincial and national estimates for the coverage of seven maternal and child health services from the MICS 2003 in Afghanistan. We compared the weighted and unweighted estimates to study the effect of these sampling weights on bias and precision of survey estimates and discuss the implications for baseline assessment and evaluation of health programmes.

## MATERIALS AND METHODS

### MICS 2003 methodology

#### Sampling frame

The target population for this study was the settled population of Afghanistan living in 32 provinces according to the 1979 census. [Provinces of Panjsher and Daykundi were created after the MICS 2003.]

#### Sample-size and sample design

The indicators relating to vaccinations required the largest sample-size. The smallest target group for these indicators was children aged 12-23 months. An earlier MICS conducted in the eastern region of Afghanistan estimated that an average of 0.26 children aged 12-23 months lived in each household (12). The survey planners concluded that a precision level of ±10% of the estimated prevalence was desired at the provincial level. With these specified, assuming a design effect of 1.5 and a prevalence of 50%, the needed sample-size was 138-144 children aged 12-23 months in every province, which would be met by surveying 550 households in every province. Under the standard assumption of the above parameters being constant, the sampling error would be lower for indicators where the target age-group was wider, e.g. supplementation of vitamin A for children aged 6-59 months.

A stratified multi-stage cluster-sampling design was used for the sampling of households in the 32 provinces, where each province was a stratum. In each of the 32 provinces, a cluster was a village or a town. Information on the number of households in each village and town of every province was collected from the 1979 census database. In total, 20 clusters were systematically selected without replacement in each province with probability of selection being proportional to size (PPS), where size was the number of households in a cluster. Villages and towns (*mahals*) with their number of households were listed in geographical order, and from cumulative households, after a random start, subsequent clusters were selected after a fixed interval. These clusters were specified as the primary sampling units (PSUs). To collect information on 550 households per province, the total number of households surveyed in every cluster ranged from 27 to 28. The 32 provinces were included as 32 strata during analysis of data.

Clusters initially inaccessible for reasons, such as flood or absence of village head, were covered at a later date. Only one cluster in one province could not be reached for security reasons (a clash between two rival villages). No cases where one selected cluster had split into two or more clusters were encountered during the survey. In each sampled cluster, the number and location of households were verified with the elderly local residents, and a sketch-map indicating well-known landmarks, such as mosques, schools, and health centres, was prepared. In cases where a selected cluster from the 1979 list had been destroyed by war, it was replaced by the next cluster from the household listing. The replacement rate was less than 10% in all the provinces. Clusters that emerged after 1979 could not be selected since the 1979 census list was used as the sampling frame.

### Selection of households in a cluster

A household was defined as the people (men and women) usually taking their meals from the same cooking-pot and those who share household assets and accumulate their earnings to procure food and other household materials. The possibility of a dwelling/structure being inhabited by more than one household was considered, and the surveyors were instructed to count each household separately in such cases. Every sampled cluster was partitioned into segments of approximately 55 households each, and one segment was randomly selected. All the households in the selected segment were listed separately even if they lived in the same structure, such as an apartment house or multi-family compound, and every alternate household was interviewed with a random start (1st or 2nd). If a selected household was absent on the day of interview, up to two additional efforts were made on later dates. In cases where no interview could be conducted after three attempts, the selected household was replaced by the nearest household next door. Data were collected for all the respondents meeting the eligibility requirements for an indicator in a sampled household. Households where eligible res-pondents refused to participate in the survey were replaced by the nearest household next door.

### 2004 pre-census data-collection

During 2004, the Central Statistics Office (CSO) of Afghanistan sent teams to conduct door-to-door enumeration in all the 32 provinces. In 29 provinces, complete enumeration was conducted, and in three provinces with areas where conditions were deemed too dangerous to send field workers, only partial enumeration was possible. This pre-census laid the ground work for future censuses by providing codes for each province, district, village, sub-village (in large villages), urban sector (*nahia*), and block. Households were also numbered and counted. Standardized quality-assurance procedures were followed, including several layers of supervisory teams and systematic re-collection of data from selected sites to ensure consistency. Based on this work, the CSO published the official census figures for all the provinces in 2006 (3). While the figures for 29 provinces were based on complete enumeration, the figures for three unsecure provinces were based on partial enumeration supplemented by projections based on demographic models.

### Generation of sampling weights based on 2004 pre-census

Although it was designed to be self-weighted, the MICS 2003 sample could not be considered self-weighted. There were significant changes in number and distribution of households in the country during 1979-2004 due to displacement and growth of population over time. The list of villages and towns based on the 1979 census was outdated and incomplete as new villages had come into existence while some villages had been displaced due to war and natural disasters, such as floods and draughts (13). Therefore, the sampling design was used for generating sampling weights in the present study. The sampling weight for every sampled household in a province was the inverse of the selection probability of that household.

The formula to generate the sampling weight for a household (h) in sampled segment (i) within the sampled cluster (k) in province P was as follows:

W_pih_=1/[(a_p_/a_j_)*(1/b_pk_)*(c_pih_/c_pil_)] [1]

where

a_p_=Number of primary sampling units (PSUs) selected in province P;

a_j_=Number of PSUs in province P;

b_pk_=Number of segment(s) in a selected PSU k in province P;

c_pih_=Number of households selected in a selected segment i in PSU k in province P;

c_pil_=Number of households in a selected segment i in PSU k in province P;

The W_pih_ value for each household was used as its sampling weight for provincial estimates.

The additional factor for a household (h) in province P to generate the national estimate was as follows:

I_ph_=[(∑N_ph_)/N_ph_] [2]

where

N_ph_=Total number of households in province P

The formula to generate sampling weight for national estimates was as follows:

W_pih_^n^=[W_pih_* I_ph_] [3]

The W_pih_^n^ value for each household was used as its sampling weight for national estimates.

The provincial and national sampling weights were normalized to sum to the sample-size. The two provinces—Panjsher and Daykundi—were created after the 2003 MICS from Parwan and Uruzgan respectively. The 2006 census figures for Panjsher and Daykundi were combined with Parwan and Uruzgan respectively. These figures were then used for generating sampling weights for Parwan and Uruzgan.

The SVYTAB command in the Stata software was used for the calculation of variance estimates taking the design of the survey into account (14). By default, the SVY set of commands compute standard errors using a linearized variance estimator based on a first-order Taylor series approximation (15). In the non-survey context, this variance estimator is referred to as the robust variance estimator (Huber-White sandwich estimator). Each province was specified as the stratum and each cluster as the PSU. The weighted estimates and confidence intervals were calculated by specifying sampling weights in the SVY command. The reported indicators were proportions which used total numbers of women or children as denominators. Since these were not fixed for a given province but are random variables, we estimated the variance of a ratio. This estimation is done automatically when this type of analysis is specified in the Stata program. For proportions, the confidence interval was derived using a logit transformation so that the interval lies between 0 and 1 (16).

The UNICEF defined the coverage variables as the proportion of the population not covered so that a higher point estimate represents a worse situation, i.e. lower coverage. We retained these definitions to be comparable with the original MICS 2003 report published by the UNICEF (2). The difference between weighted and unweighted point estimates and confidence intervals was calculated by subtracting unweighted estimates from weighted estimates. We also calculated the range, mean, median, and absolute mean of the differences for each of the seven indicators.

The following three indicators describe the coverage of health services for women: (a) percentage of women, aged 15-49 years, who delivered in the past two years before the survey and were attended during delivery by unskilled health personnel, i.e. excluding doctor, nurse, or midwife; (b) percentage of women currently married or in union, aged 15-49 years, who were not using a contraceptive method; and (c) percentage of women, aged 15-49 years, who delivered in the past two years before the survey and received antenatal care only from unskilled health personnel, i.e. excluding doctor, nurse, or midwife.

The following four indicators provide information on the coverage of health services to children: (a) percentage of children, aged 6-59 months, who did not receive at least one high-dose vitamin A supplement in the last six months; (b) percentage of children, aged 9-59 months, who were not immunized against measles; (c) percentage of children, aged 12-23 months, who did not receive three doses of DPT immunization; and (d) percentage of children, aged less than five years, who did not receive BCG immunization.

### Ethical issues

Although a formal ethics committee did not exist in Afghanistan to review the MICS questionnaire, representatives from the MoPH, Ministry of Rural Rehabilitation and Development, Kabul University, international agencies, and non-governmental organizations were involved in the technical review of the entire questionnaire and survey methodolo-gy. Voluntary consent was taken at the beginning of the questionnaire by the interviewer who read out the statement before administering the questionnaire.

## RESULTS

### Comparison of weighted and unweighted point estimates

Among the three indicators relating to the coverage of health services for women, the widest range of differences between weighted and unweighted point estimates was for the percentage of deliveries conducted by unskilled birth attendants. The difference ranged from −13.35 (Samangan) to 5.21 (Badghis), with a difference in the national estimate of 1.77 (Table 1). Among the four indicators relating to the coverage of health services for children, the widest range of differences between weighted and unweighted estimates was for the percentage of children, aged less than five years, who did not receive BCG immunization. The difference ranged from −16.5 (Faryab) to 17.91 (Takhar), with a difference in the national estimate of 0.79.

**Fig. 1.**
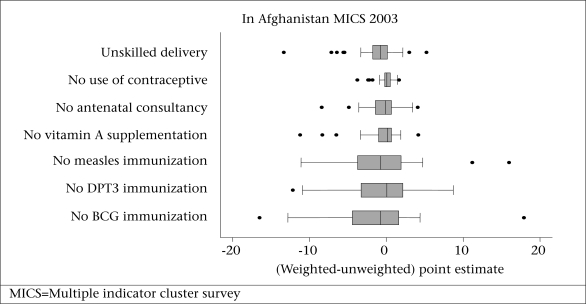
Boxplot of differences in point estimates

Across all the provinces, the median difference between weighted and unweighted point estimates was close to zero for every indicator (Fig. 1). The interquartile range of differences for the four indicators relating to the coverage of health services to children was wider than the three indicators relating to the coverage of health services to women. In total, more than 90% of unweighted estimates were within 10 percentage points of weighted estimates (Table 1). The direction of difference between weighted and unweighted point estimates ranged from a high of 65% values negative for delivery by unskilled birth attendant to a low of 35% values negative for couples not using a method to delay pregnancy. The average difference (weighted unweighted) across the seven indicators ranged from −1.52 to −0.06 percentage points, and the average absolute difference ranged from 0.75 to 4.07 percentage points. The difference in national point estimates ranged from −1.82 to 2.19 percentage points across the seven indicators.

The provinces were ranked for each indicator based on the weighted and unweighted point estimates, and the provinces with the five highest and the five lowest values were compared. The provinces included among the five highest and the five lowest were similar, although the comparative ranking within the groups of five was not identical. Four of five provinces were the same for all indicators, except the indicator on delivery by unskilled attendants, where only three lowest ranked provinces were the same. Both weighted and unweighted point estimates reflected a relatively better situation for children compared to women in Afghanistan.

### Comparison of weighted and unweighted confidence intervals

Among the three indicators relating to the coverage of health services for women, the widest difference between weighted and unweighted confidence intervals was for the percentage of deliveries conducted by unskilled birth attendants (Table 2). The difference in confidence intervals ranged from −5.68 (Ghazni) to 20.67 (Paktya), with a mean of 2.91, an absolute mean of 3.52, and a median of 1.97. Among the four indicators relating to the coverage of health services for children, the widest range of the difference between weighted and unweighted confidence intervals was for the percentage of children, aged less than five years, who did not receive BCG immunization. The difference ranged from −15.34 (Faryab) to 15.9 (Takhar), with a mean of 1.14, an absolute mean of 4.55, and a median of 0.94.

**Fig. 2.**
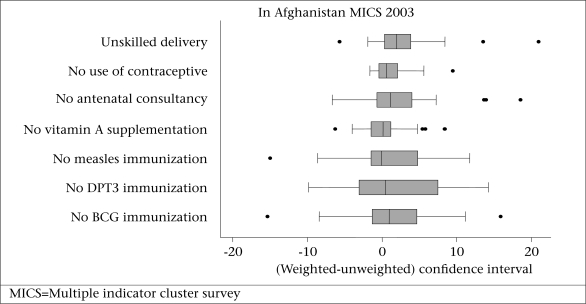
Boxplot of differences in confidence intervals

The median difference between weighted and unweighted confidence intervals ranged from −0.04 to 1.97 for the seven indicators looking across all the provinces (Fig. 2). The interquartile range of differences for the four indicators relating to the coverage of health services for children was wider than the three indicators relating to the coverage of health services for women. In total, more than 90% of unweighted confidence intervals were within 10 percentage points of weighted confidence intervals (Table 2). The direction of difference between weighted and unweighted point estimates ranged from a high of 50% values negative for children aged 9-59 months not receiving measles immunization to a low of 15% values negative for delivery by unskilled birth attendant. The average difference (weighted unweighted) across the seven indicators ranged from 0.34 to 2.91 percentage points, and the average absolute difference ranged from 1.67 to 5.67 percentage points. The difference in national confidence intervals ranged from 0.34 to 2.47 percentage points.

**Table 1.** Weighted point estimates and the difference between weighted and unweighted point estimates for seven health service-coverage indicators from the 2003 Multiple Indicator Cluster Survey of Afghanistan

Province	Last delivery assisted by unskilled birth attendant (in the last 2 years)	Wt-unwt	Married woman aged >50 years currently not using a method to delay pregnancy	Wtunwt	Antenatal consultation not taken from doctor/ TBA during the last pregnancy	Wt-unwt	Children, aged 6-59 months, who did not receive vitamin A supplementation	Wtunwt	Children, aged 9-59 months, who did not receive measles immunization	Wtunwt	Children, aged 12-23 months, who did not receive 3 doses of DPT immunization	Wtunwt	Children, aged 5 years, who did not receive BCG immunization	Wtunwt
Badakshan	96.93	-1.55	97.04	-0.61	95.50	-0.83	2.90	0.21	12.81	-1.27	58.36	-0.78	17.42	-0.92
Badhgis	93.61	5.21	97.96	-0.84	99.86	0.68	5.78	0.41	40.36	-2.50	85.11	-10.60	66.24	-12.79
Baghlan	93.54	-0.99	94.60	1.32	92.67	-3.10	34.83	-6.49	44.05	-6.13	93.91	1.80	79.18	-0.48
Balkh	95.27	-0.75	94.23	-0.17	96.88	-0.24	2.15	-1.53	28.48	-2.93	75.90	-1.02	40.64	-4.27
Bamiyan	89.15	-3.30	94.07	0.06	92.54	-0.58	16.12	-0.11	24.22	-8.53	97.47	-0.53	56.23	-0.69
Farah	80.80	-7.15	73.06	-2.21	96.45	2.12	7.72	0.27	18.78	-2.67	66.25	-5.54	35.44	-6.01
Faryab	95.92	-1.94	95.17	-0.07	87.94	-1.67	17.48	-11.22	20.80	-11.11	54.90	-12.17	28.73	-16.50
Ghazni	95.77	3.00	98.10	1.04	89.05	3.46	46.24	4.16	28.71	4.02	82.89	-0.44	35.21	1.52
Ghor	90.70	0.00	99.20	0.00	99.23	0.00	15.60	0.00	47.62	0.00	94.38	0.00	53.15	0.00
Helmand	97.33	-1.04	98.90	0.68	89.52	-4.89	5.38	-3.35	9.97	-3.63	98.06	0.13	37.18	-10.09
Heart	83.89	2.15	79.41	1.67	83.73	-1.56	6.27	-0.44	20.89	-2.17	57.06	-8.94	27.61	-7.24
Jawzjan	91.94	1.29	97.09	0.37	90.90	1.28	6.97	0.28	22.43	2.10	85.79	4.43	57.18	0.73
Kabul	74.23	-5.59	84.57	-1.79	71.86	-2.18	6.71	-0.01	20.65	2.64	32.30	-8.11	23.11	-4.58
Kandhar	96.41	-1.48	88.20	-3.76	99.13	-0.26	5.47	-3.10	32.03	1.52	98.33	1.49	77.38	1.48
Kapisa	81.34	-6.42	86.18	-2.36	77.56	-8.40	20.46	1.82	34.45	11.19	81.05	-2.28	69.84	-3.69
Khost	83.50	1.31	98.31	0.07	85.92	0.70	23.18	0.80	27.48	0.05	77.32	1.73	20.40	-0.72
Kunar	97.11	0.18	99.78	0.32	96.89	-0.01	7.96	-0.61	7.57	-0.50	35.48	0.86	16.53	-2.09
Kunduz	95.56	-0.80	94.99	0.66	96.58	0.63	65.41	1.87	71.25	-0.94	96.61	1.57	67.60	-1.08
Laghman	86.83	-0.55	91.79	0.27	79.73	0.00	7.56	-1.73	15.81	0.00	55.95	8.54	17.21	3.16
Logar	89.90	-1.43	87.11	-0.90	73.11	-0.20	5.37	0.57	6.60	1.89	38.57	-4.29	14.14	2.61
Nangarhar	87.75	-2.20	94.72	1.09	86.20	-1.27	5.50	0.17	11.12	1.94	28.35	4.21	14.81	-1.74
Nimroz	93.07	0.19	87.17	1.46	94.49	4.08	9.81	0.15	45.12	15.97	77.97	8.74	38.62	4.41
Nooristan	98.36	-0.28	99.72	0.29	98.07	0.10	35.45	0.58	32.34	0.96	86.58	7.73	51.86	1.69
Paktika	95.39	0.00	99.87	0.34	97.08	-0.30	4.20	0.30	17.90	-1.49	93.54	5.11	32.47	2.92
Paktya	85.68	-5.46	97.49	0.20	90.85	-3.57	10.47	-0.47	25.38	-6.16	51.04	-10.92	26.91	-4.82
Parwan	94.84	-1.43	93.14	0.00	90.71	-3.02	15.28	1.28	35.00	3.17	81.76	-0.65	56.87	1.78
Samangan	57.77	-13.35	97.37	0.84	97.45	2.25	7.58	-8.31	12.34	-6.65	92.49	2.49	56.15	-7.64
Saripol	99.45	-0.10	95.67	-0.27	96.44	-1.30	9.70	-1.40	12.80	-3.87	83.44	-0.98	52.42	-0.82
Takhar	99.59	0.28	99.64	0.35	97.82	2.05	7.71	0.88	8.99	-6.93	96.81	5.23	63.45	17.91
Uruzgan	94.27	-0.06	96.52	-0.02	99.07	0.30	30.29	0.77	42.36	-6.11	96.83	0.43	78.60	0.24
Wardak	88.01	-1.15	94.84	0.02	90.26	0.54	9.01	1.45	25.70	4.74	73.49	-4.56	16.95	2.34
Zabul	99.14	0.07	98.21	0.00	99.14	0.08	17.97	-0.32	45.25	0.15	94.88	1.00	47.53	-3.50
Mean		-1.35		-0.06		-0.47		-0.72		-0.73		-0.51		-1.53
Absolute mean		2.21		0.75		1.61		1.72		3.87		3.98		4.08
Median		-0.78		0.06		-0.10		0.16		-0.72		0.06		-0.77
National	87.49	1.77	91.20	1.46	86.11	2.19	12.63	-1.82	23.71	-0.26	71.07	1.15	41.00	0.79

TBA=Traditional birth attendant;

Wt-unwt=Weighted estimate minus unweighted estimate

**Table 2.** Weighted confidence intervals and difference between weighted and unweighted confidence intervals for seven health service-coverage indicators from the 2003 Multiple Indicator Cluster Survey of Afghanistan

Province	Last delivery assisted by unskilled birth attendant (in the last 2 years)		Married woman aged <50 years currently not using a method to delay pregnancy	Wtunwt	Antenatal consultation not taken from doctor/ TBA during the last pregnancy	Wtunwt	Children, aged 6-59 months, who did not receive vitamin A supplementation	Wtnwt	Children, aged 9-59 months, who did not receive measles immunization	Wtunwt	Children, aged 12-23 months, who did not receive 3 doses of DPT immunization	Wtunwt	Children, aged <5 years, who did not receive BCG immunization	Wtunwt
Badakshan	5.40	2.00	5.07	1.53	15.38	7.04	2.79	-1.69	15.21	-0.69	27.06	-1.30	13.08	0.56
Badhgis	22.43	5.61	2.49	0.15	1.33	-4.44	6.79	-0.56	22.34	-3.84	24.73	13.63	25.34	8.59
Baghlan	13.07	6.59	6.78	0.89	25.06	13.67	32.42	5.81	30.41	4.41	12.36	-3.84	20.94	0.07
Balkh	8.27	2.42	6.80	0.06	8.59	-0.14	4.62	-2.01	24.62	5.50	19.83	-2.15	23.85	5.08
Bamiyan	7.74	1.76	5.96	0.16	12.67	4.40	21.82	8.39	28.13	9.44	11.16	3.97	27.46	4.72
Farah	10.29	0.34	22.12	9.49	6.57	0.31	5.69	-0.35	13.53	-2.23	39.77	11.18	18.26	-6.08
Faryab	6.67	2.73	6.20	1.96	14.11	3.62	26.97	-6.26	17.76	-14.99	22.38	-9.83	19.16	-15.34
Ghazni	9.60	-5.68	2.69	-1.05	14.74	-6.68	16.79	0.95	24.11	8.18	19.39	6.97	19.80	3.95
Ghor	15.55	0.00	3.50	0.00	2.84	0.00	14.84	0.00	28.00	0.00	14.07	0.00	27.51	0.00
Helmand	9.72	3.69	2.52	-0.64	32.31	18.55	9.65	-3.99	13.14	-1.46	7.42	1.65	26.60	-0.26
Herat	15.32	-1.15	14.24	1.61	16.64	0.47	6.37	0.25	18.01	-5.15	45.65	13.85	19.45	-8.42
Jawzjan	13.48	4.02	10.10	4.07	9.83	1.13	9.21	1.29	13.43	-0.08	20.13	-5.25	15.58	-2.10
Kabul	26.82	13.55	13.86	2.25	9.63	-2.76	8.19	-0.70	28.83	10.12	37.21	11.65	27.83	7.47
Kandhar	6.86	3.40	8.19	2.77	3.92	1.74	8.76	-1.21	24.48	-1.34	5.24	-3.90	21.58	4.18
Kapisa	24.94	6.88	11.97	2.80	23.68	6.89	13.79	0.94	25.83	7.53	34.17	14.24	30.11	8.01
Khost	14.94	1.35	2.12	0.01	13.85	1.69	15.28	-3.98	20.76	-1.01	20.07	1.73	18.47	0.55
Kunar	8.38	0.73	0.77	-0.68	6.47	1.03	10.14	0.49	8.59	-1.06	29.07	-0.77	9.24	-4.53
Kunduz	10.08	4.31	15.05	5.61	4.80	-2.18	26.27	-2.80	18.88	0.52	8.99	-7.32	27.61	2.28
Laghman	16.44	1.95	11.34	1.54	14.81	2.30	7.90	-2.70	11.17	-1.02	43.04	11.20	25.41	11.16
Logar	9.65	2.60	13.29	4.45	10.71	-0.88	3.28	-0.54	7.04	3.05	16.21	-6.46	10.93	2.64
Nangarhar	12.08	2.89	11.12	2.55	19.61	1.79	4.30	0.95	15.75	5.20	29.73	10.57	14.67	0.30
Nimroz	12.31	1.63	11.51	-1.61	10.44	-6.28	10.58	1.09	38.77	11.73	24.05	-0.70	30.12	4.63
Nooristan	4.70	0.91	0.97	-0.47	6.72	1.10	34.56	5.40	32.44	3.19	21.06	-6.40	20.34	-7.47
Paktika	5.24	0.36	0.50	-0.73	5.36	2.26	5.13	0.97	14.71	2.87	9.55	-0.60	14.51	1.32
Paktya	34.38	20.97	4.04	0.35	24.97	13.95	9.55	-0.47	16.96	1.51	32.40	6.08	15.39	-0.89
Parwan	8.24	3.08	7.63	-0.87	15.35	7.25	10.74	0.27	19.63	-1.47	24.33	0.32	21.98	2.74
Samangan	24.56	8.44	7.04	1.43	9.92	1.12	21.79	8.44	11.61	-2.22	24.53	8.02	21.05	-7.67
Saripol	3.80	0.72	5.69	1.79	10.39	5.22	10.32	-3.80	8.49	-8.61	23.14	2.44	24.51	1.44
Takhar	1.80	-0.54	1.12	-0.39	6.50	-2.08	9.78	-0.62	10.62	-7.00	9.91	-6.83	42.03	15.90
Uruzgan	10.18	-1.88	3.95	-0.46	2.62	-0.42	19.96	1.47	28.83	-0.11	7.56	0.66	16.54	-1.76
Wardak	6.99	-0.56	6.60	0.99	7.05	-1.26	11.11	4.73	20.88	7.03	22.78	6.70	9.92	0.07
Zabul	2.46	0.19	2.53	-0.38	3.18	-0.48	17.09	1.33	28.71	2.93	10.16	-2.33	32.15	5.56
Mean		2.92		1.22		2.12		0.35		0.97		2.10		1.15
Absolute mean		3.53		1.68		3.85		2.33		4.23		5.70		4.55
Median		1.97		0.63		1.11		0.12		-0.04		0.49		0.94
National	4.16	1.40	2.28	0.34	4.21	1.36	3.03	0.53	4.88	1.24	7.31	2.47	5.47	1.57

Wt-unwt=Weighted estimate minus unweighted confidence interval

The provinces were ranked for each indicator based on the confidence intervals, and the provinces with the five widest and the five narrowest values were compared. In total, four provinces included among the five narrowest were the same for all the indicators, except for unskilled birth attendants at delivery and lack of measles immunization, where only three provinces were the same. There was greater heterogeneity between the rankings of the five provinces with the widest values of the confidence intervals. On average, three provinces were different for each indicator, with up to five provinces different for BCG immunization.

## DISCUSSION

Estimates generated using sampling weights were unbiased compared to unweighted estimates. The use of sampling weights is a widely-agreed method for descriptive analyses as it adjusts the sample to be representative of the population from which it is derived (8,9). In this study, data were collected in 2003 but with a sampling frame from 1979. The sampling weights generated based on the 2004 pre-census data improved the generalizability of the results for the population living in Afghanistan in 2003.

The use of sampling weights generated from the data on distribution of villages and household popu-lations in the 2004 pre-census allowed reduction in bias and adjustment of precision in estimates. This study provided a unique opportunity to measure the bias that can arise from using an outdated sampling frame for estimating baseline measures of the coverage of health services in post-conflict countries. The use of sampling weights leads to larger variances and, thus, widening of confidence intervals. In the present study, we found that the sampling weights were associated with differences in point estimates and confidence intervals for provincial and national estimates. Comparison of weighted and unweighted estimates resulted in some wide differences in magnitude for individual provincial estimates and confidence intervals but, in general, these differences did not lead to different conclusions about the cross-sectional point estimates made at the national level. The mean, absolute mean, and median of difference between weighted and unweighted estimates and confidence intervals were close to zero for all the indicators.

The MICS 2003 was originally intended to generate estimates for children aged 12-23 months, with a precision level of ±10% (of estimated prevalence) at the provincial level (2). Of the seven indicators analyzed in this study, the indicator on DPT immunization was directly related to this age-group. The study found that more than 50% (19 of 32) of weighted estimates for this indicator had a precision level lower than the intended level of ±10% (of estimated prevalence). Confidence intervals wider than 20 percentage points were also found for other coverage indicators. All the indicators included in the study were gathered for descriptive analysis of coverage of health services and to estimate the proportion of individuals in the population who have a certain characteristic. The widening of confidence intervals is unfortunate but not critical because the weighted estimates with confidence intervals offer a valid description of the population and estimators for the baseline assessment of coverage of health services in post-conflict Afghanistan. However, since the variances are higher than originally anticipated, policy-makers will need to have a higher tolerance for error in assessing future change. The MICS 2003 will allow policy-makers to make plausible inferences about future changes in health services but they may not reach the probability levels frequently expected in scientific research (17).

The ranking of the five highest and the five lowest provinces on weighted and unweighted estimates and confidence intervals also yielded similar results. For management and evaluation purposes, this allows stakeholders to appropriately identify which provinces needed the most improvement, and where extra effort is needed. The Government has continued to emphasize expanding the coverage of basic health services represented by these coverage indicators and, by using additional data on the quality of services through a Balanced Scorecard, has focused on improving the quality of these services, especially in provinces where there are deficiencies (18).

The general consistency of the results calculated with and without the sampling weights suggests that outdated sampling frames may be acceptable for use in similar contexts to obtain baseline estimates from household surveys to guide policy decisions, although at a lower level of statistical probability than originally planned. However, this conclusion may not be generalizable to other similar settings because of specific characteristics of the MICS 2003. First, data of the MICS 2003 were collected using a probability-based sampling technique in a scientifically-rigorous manner to keep the replacement rate for selected clusters low (below 10% in all provinces). Second, clusters created after 1979 had a zero probability of selection in the MICS 2003 sample. In this study, data from these clusters were used for generating the sampling weights and calculate the weighted estimates and confidence intervals. The use of weights to adjust for these clusters involves the assumption of homogeneity across clusters created before and after 1979. A difference in characteristics between these two groups of clusters would violate this assumption and bias the weighted estimates. Two useful techniques could have been used for testing for this: First, aerial photographs of villages to cross-check and supplement the listing of households available in the 1979 census and subsequent use of these updated lists for sampling (19); second, a similar survey from a representative sample of households right after the 2004 pre-census. The aerial photograph technique was used for the 1972 Demographic and Family Guidance Survey of the settled population of Afghanistan (19). There was no national census conducted in Afghanistan before the 1972 survey. Aerial photographs supplemented the information available from (a) topographic series maps and (b) lists of villages with crude population estimates. These photographs were used for household prelisting, boundary marking, sampling, and quality control re-interviewing. Unfortunately, neither of these techniques was possible at the time of MICS in 2003. In such situations, the use of sampling weights derived from sources of information available later in time is a pragmatic choice to correct the bias in the health service-coverage indicators due to outdated sampling frames.

## ACKNOWLEDGEMENTS

The study was funded by a contract with the Afghanistan Ministry of Public Health and the Johns Hopkins University Bloomberg School of Public Health, in collaboration with the Indian Institute of Health Management Research. The authors thank the staff who participated in data-collection for the MICS in 2003 and CSO pre-census in 2004.
